# Diminished Antimicrobial Peptide and Antifungal Antibiotic Activities against *Candida albicans* in Denture Adhesive

**DOI:** 10.3390/antibiotics6010006

**Published:** 2017-02-06

**Authors:** Amber M. Bates, Jorge L. Garaicoa, Carol L. Fischer, Kim A. Brogden

**Affiliations:** 1 Iowa Institute for Oral Health Research, College of Dentistry, The University of Iowa, Iowa City, IA 52242, USA; amber-bates@uiowa.edu; 2 Department of Restorative Dentistry, School of Dentistry, Oregon Health and Science University, Portland, OR 97201, USA; garaicoa@ohsu.edu; 3 Department of Biology, Waldorf University, Forest City, IA 50436, USA; Carol.Fischer@waldorf.edu

**Keywords:** denture adhesive, *Candida albicans*, antimicrobial peptides, antifungal antibiotics, radial diffusion assay

## Abstract

The underlying causes of denture stomatitis may be related to the long-term use of adhesives, which may predispose individuals to oral candidiasis. In this study, we hypothesize that antimicrobial peptides and antifungal antibiotics have diminished anti-*Candida* activities in denture adhesive. To show this, nine antimicrobial peptides and five antifungal antibiotics with and without 1.0% denture adhesive were incubated with *Candida albicans* strains ATCC 64124 and HMV4C in radial diffusion assays. In gels with 1.0% adhesive, HNP-1, HBD2, HBD3, IP-10, LL37 (only one strain), histatin 5 (only one strain), lactoferricin B, and SMAP28 showed diminished activity against *C. albicans*. In gels with 1.0% adhesive, amphotericin B and chlorhexidine dihydrochloride were active against both strains of *C. albicans*. These results suggest that denture adhesive may inactivate innate immune mediators in the oral cavity increasing the risk of *C. albicans* infections, but inclusion of antifungal antibiotics to denture adhesive may aid in prevention or treatment of *Candida* infections and denture stomatitis.

## 1. Introduction

*Candida* species infections can cause complications for individuals with dentures, such as denture stomatitis. In a previous study, broth microdilution assays were used to show that antimicrobial peptides (AMPs) lactoferricin B and sheep myeloid antimicrobial peptide 28 (SMAP28) and antimicrobial lipids sphingosine, dihydrosphingosine, and phytosphingosine in 1.0% denture adhesive lost antimicrobial activity against *Candida albicans* (*p* < 0.05), but amphotericin B (AMB), chlorhexidine dihydrochloride (CHX), chlorhexidine gluconate (CHG), fluconazole (FLC), and nystatin (NYT) in 1.0% denture adhesive did not [1]. However, there were limitations of the broth microdilution assay that impeded the development of the concept. First, large concentrations (~100.0 µg) of AMPs, antimicrobial lipids, and antifungal antibiotics were needed in the broth microdilution assays. Second, the diluted denture adhesive used was very difficult to mix in 96-well plates due to its thick viscosity. Third, a precipitate often would form when the AMPs were mixed with the denture adhesive [1]. Therefore, a radial diffusion assay [2] was used to test additional AMPs and antifungal antibiotics to further develop these concepts.

The objective of this study was to determine the activity of AMPs and antifungal antibiotics in gels with and without 1.0% denture adhesive against *C. albicans* strains ATCC 64124 and HMV4C.

## 2. Results

### 2.1. AMP Activity

HNP-1, HBD2, HBD3, IP-10, LL37, SMAP28, lactoferricin B, and histatin 5 had diminished activity against *C. albicans* HMV4C in gels with 1.0% denture adhesive (Table 1). HBD-1 had a minimal inhibitory concentration (MIC) > 1000.0 µg/mL (e.g., >254.1 µM) in both gels with or without 1.0% denture adhesive. HNP-1, HBD2, HBD3, IP-10, SMAP28, and lactoferricin B had diminished activity against *C. albicans* ATCC 64124 in gels with 1.0% denture adhesive (Table 1). HBD-1, LL37, and histatin 5 had MIC > 1000.0 µg/mL (e.g., >254.1 µM, >222.4 µM, and >329.0 µM, respectively) in both gels with or without 1.0% denture adhesive.

### 2.2. Antifungal Antibiotics

NYT had diminished (*p* < 0.05) antimicrobial activity against *C. albicans* ATCC 64124 and HMV4C in gels with 1.0% denture adhesive (Table 1). CHG had diminished antimicrobial activity against *C. albicans* ATCC 64124 in gels with 1.0% denture adhesive, but CHG had the same antimicrobial activity against *C. albicans* HMV4C with and without 1.0% denture adhesive present. AMB, CHX, and FLC had the same (*p* > 0.05) antimicrobial activity against *C. albicans* ATCC 64124 and HMV4C in gels with and without 1.0% denture adhesive. However, the MIC for FLC was not detected in the ATCC 64124 strain (>1000.0 µg/mL, 3265.1 µM).

## 3. Discussion

Recently, Garaicoa and colleagues determined that AMPs and antimicrobial lipids endogenous to the oral cavity showed diminished activity when added to 1.0% denture adhesive suggesting that components in adhesives may inactivate local innate immune factors possibly predisposing denture wearers to *Candida* species infections [1]. More importantly, antifungal antibiotics retained their anti-*C. albicans* activity in denture adhesive strongly suggesting that antifungal antibiotics could be candidates for inclusion in adhesive formulations and used as prescribed topical treatments for individuals with denture stomatitis [1]. In this study, we extend these findings and show that numerous AMPs found in the oral cavity have diminished activity in 1.0% adhesive. Radial diffusion assays were used to assess the activity of nine AMPs and five antifungal antibiotics with and without 1.0% denture adhesive to *C. albicans* strains ATCC 64124 and HMV4C. In gels with 1.0% adhesive, HNP-1, HBD2, HBD3, IP-10, LL37 (only one strain), histatin 5 (only one strain), lactoferricin B, and SMAP28 showed diminished activity against *C. albicans*. In gels with 1.0% adhesive, AMB and CHX were active against both strains of *C. albicans*. These results suggest that denture adhesive may inactivate innate immune mediators in the oral cavity increasing the risk of *C. albicans* infections, but inclusion of antifungal antibiotics to denture adhesive may aid in prevention or treatment of *Candida* infections and denture stomatitis.

Several AMPs that would normally inhibit the growth of *C. albicans* did not inhibit growth in 1.0% denture adhesive. This suggests that denture adhesive may inhibit activity of innate immune mediators in the oral cavity, therefore increasing the risk of fungal infections. It also suggests that the use of denture adhesive may positively correlate with the incidence of denture stomatitis. AMP activity is diminished in environments that contain serous proteins, increased concentrations of sodium chloride, or altered pH [3]. It is likely that AMP activity is diminished by components in the denture adhesive. Although this study only examined antimicrobial activity in 1.0% denture adhesive, it is likely that there is greater inhibition of these AMPs in full denture adhesive. To what extent this is occurring in the oral cavity to individuals using denture adhesives is not yet known.

Inclusion of antifungal antibiotics to denture adhesive may aid in prevention of fungal infections. From this study, AMB and CHX appear to be good candidates for inclusion as their anti-*C. albicans* activity was not affected by the presence of denture adhesive in either strain. Future research should look into how the function of AMPs is being inhibited by the adhesive and the role of this in *Candida* infections.

## 4. Materials and Methods

### 4.1. C. albicans, Denture Adhesive, AMPs, and Antifungal Antibiotics

*C. albicans* strains ATCC 64124 and HMV4C were used and cultivated as previously described [1]. Their identities were verified (State Hygienic Laboratory, University of Iowa Research Park, Coralville, IA, USA). These cultures were adjusted to contain 1.2–3.7 × 10^6^ CFU/mL in Roswell Park Memorial Institute (RPMI) 1640 medium.

The denture adhesive used was a standard, over-the-counter gel containing poly sodium-calcium mixed partial salt, petrolatum, cellulose gum, and mineral oil and was prepared as previously described [1]. Denture adhesive was too viscous to use directly and was diluted to 1.0% for incorporation into the radial diffusion assay.

Nine AMPs were used. Sheep myeloid antimicrobial peptide 28 (SMAP28, NeoMPS, Inc., San Diego, CA, USA) was used as a control because of its known antimicrobial activity against *C. albicans* [4]. Human neutrophil peptide-1 (HNP-1, Peprotech, Rocky Hill, NJ, USA), recombinant human CXCL10 (IP-10, Peprotech, Rocky Hill, NJ, USA), lactoferricin B (AnaSpec, Inc., Fremont, CA, USA), histatin 5 (AnaSpec, Inc., Fremont, CA, USA), human β defensin-1 (HBD-1, Peprotech), human β defensin-2 (HBD2, Peprotech), human β defensin-3 (HBD3, Peprotech), and LL37 (AnaSpec) were used.

Five antifungal antibiotics were used. These included FLC (Sigma-Aldrich, St. Louis, MO, USA), NYT (Sigma-Aldrich), CHG (GUM, Sunstar Americas Inc., Schaumburg, IL, USA), AMB (Sigma-Aldrich), and CHX (Sigma-Aldrich).

The AMPs and antifungal antibiotics were serially diluted to determine the MIC, ranging from 200.0 µg/mL to 0.0 µg/mL, and for some 1000.0 µg/mL to 0.0 µg/mL. Each agent was reconstituted in distilled water with 0.01% acetic acid for resuspension to keep the agent in solution. Two-fold dilutions were prepared.

### 4.2. Radial Diffusion Assay

The radial diffusion assays were performed as described [2] with the following unique modifications. Underlay agarose gels were prepared with and without 1.0% denture adhesive in square petri dishes (Electron Microscopy Sciences, Hatfield, PA, USA). Plates without 1.0% adhesive contained 12.5 mL of 10.0 mM sodium phosphate buffer adjusted to pH 7.4, 12.5 mL of 2.0% agarose (Agarose LE, Roche Diagnostics Corporation, Indianapolis, IN, USA) in 10.0 mM sodium phosphate adjusted to pH 7.4, and 1.0 mL of *C. albicans* culture per plate. Plates with 1.0% adhesive contained 12.5 mL of 2.0% dental adhesive in 10.0 mM sodium phosphate adjusted to pH 7.4, 12.5 mL 2.0% agarose (Agarose LE, Roche Diagnostics Corporation, Indianapolis, IN, USA) in 10.0 mM sodium phosphate adjusted to pH 7.4, and 1.0 mL of *C. albicans* culture. Wells of 2.0 mm in diameter were created and filled with 5.0 µL of AMP or antifungal antibiotic dilution (*n* = three replications per test). A blank of 0.01% acetic acid in distilled water was also included. The plates were incubated at 37 °C for 3 h. After incubation, 10.0 mL of overlay containing 1.0% agarose in 10.0 mM phosphate buffer with 6.0% RPMI 1640, was added to each plate. The plates were incubated at 37 °C overnight. At 24 h, antimicrobial activity was determined by measuring the zone of clearance around the center of each well and extrapolating MIC as described [2].

### 4.3. Statistical Analysis

Results from the radial diffusion assays (*n* = 3 per test) contained MICs ranging from 1000.0 µg/mL to 0.0 µg/mL (Table 1). When MICs fell outside the range of the assay, we considered the MIC to be equal to the highest concentration used in the assay. For example, if the MIC was >1000.0 µg/mL, the MIC was conservatively estimated to be 1000.0 µg/mL for the purpose of statistical analysis. One-way fixed-effects ANOVA models were fit to the µM MIC values. Pairwise group comparisons were conducted using the method of Tukey’s Honest Significant Differences (HSD). A 0.05 level was used to determine statistically significant differences. All analyses were conducted using JMP (Version 10.0, SAS, Cary, NC, USA).

## 5. Conclusions

AMPs are present in saliva and are active against *C. albicans*. In the presence of 1.0% denture adhesive, AMPs had diminished activity against *C. albicans* strains ATCC 64124 and HMV4C. In the presence of 1.0% denture adhesive, antifungal antibiotics did not have diminished activity against *C. albicans* strains ATCC 64124 and HMV4C. Results suggest that denture adhesive may inactivate innate immune mediators in the oral cavity increasing the risk of *C. albicans* infections, but inclusion of antifungal antibiotics to denture adhesive may aid in prevention or treatment of *Candida* infections and denture stomatitis.

## Figures and Tables

**Table 1:** Minimal inhibitory concentrations (MICs) of AMPs and antifungal antibiotics against *C. albicans*. Mean (standard error, *n* = 3) values. The µM values in each row with the same letter are not significantly different (*p* < 0.05).

9 AMPs and 5 Anti-Fungal Antibiotics		*C. albicans* HMV4C		*C. albicans* ATCC 64124	
		No Adhesive	Adhesive	No Adhesive	Adhesive
Human neutrophil peptide-1 (HNP-1)	µg/mL	18.1 (0.7)	>200.0 (0.0)	142.9 (2.4)	>1000.0 (0.0)
	µM	5.2 (0.2)	>58 (0.0)	41.4 (0.7)	>290 (0.0)
		d	b	c	a
Human β defensin-1 (HBD-1)	µg/mL	>1000.0 (0.0)	>1000.0 (0.0)	>1000.0 (0.0)	>1000.0 (0.0)
	µM	>254.1 (0.0)	>254.1 (0.0)	>254.1 (0.0)	>254.1 (0.0)
		a	a	a	a
Human β defensin-2 (HBD-2)	µg/mL	15.1 (1.3)	>200.0 (0.0)	20.1 (0.7)	>200.0 (0.0)
	µM	3.5 (0.3)	>46.1 (0.0)	4.6 (0.2)	>46.1 (0.0)
		b	a	b	a
Human β defensin-3 (HBD-3)	µg/mL	15.9 (0.4)	>200.0 (0.0)	17.6 (0.8)	>200.0 (0.0)
	µM	3.1 (0.1)	>38.7 (0.0)	3.4 (0.2)	>38.7 (0.0)
		b	a	b	a
Human CXCL10 (IP-10)	µg/mL	18.6 (0.3)	>200.0 (0.0)	103.6 (3.2)	>1000.0 (0.0)
	µM	2.2 (0.0)	>23.1 (0.0)	12.0 (0.4)	>115.7 (0.0)
		d	b	c	a
Cathelicidin LL37	µg/mL	136.8 (4.5)	>1000.0 (0.0)	>1000.0 (0.0)	>1000.0 (0.0)
	µM	30.4 (1.0)	>222.4 (0.0)	>222.4 (0.0)	>222.4 (0.0)
		b	a	a	a
Sheep myeloid antimicrobial peptide 28 (SMAP28)	µg/mL	15.7 (0.1)	>200.0 (0.0)	14.4 (0.5)	>200.0 (0.0)
	µM	4.9 (0.0)	>62.5 (0.0)	4.5 (0.2)	>62.5 (0.0)
		b	a	b	a
Lactoferricin B	µg/mL	29.7 (1.1)	>1000.0 (0.0)	23.2 (0.8)	>1000.0 (0.0)
	µM	9.5 (0.4)	>320.0 (0.0)	7.4 (0.3)	>320.0 (0.0)
		b	a	c	a
Histatin 5	µg/mL	130.4 (3.0)	>1000.0 (0.0)	>1000.0 (0.0)	>1000.0 (0.0)
	µM	42.9 (1.0)	>329.0 (0.0)	>329.0 (0.0)	>329.0 (0.0)
		b	a	a	a
Nystatin (NYT)	µg/mL	9.4 (0.2)	137.2 (8.2)	9.7 (0.2)	61.0 (2.5)
	µM	10.2 (0.2)	148.1 (8.9)	10.5 (0.2)	65.9 (2.7)
		c	a	c	b
Amphotericin B (AMB)	µg/mL	5.4 (0.2)	6.2 (1.7)	6.0 (0.2)	16.6 (3.1)
	µM	5.8 (0.2)	6.7 (1.8)	6.5 (0.2)	18.0 (3.4)
		a	a	a	a
Chlorhexidine gluconate (CHG)	µg/mL	6.3 (0.1)	0.7 (0.4)	4.1 (0.2)	81.6 (3.2)
	µM	7.0 (0.1)	0.8 (0.4)	4.6 (0.2)	90.9 (3.6)
		b	b	b	a
Chlorhexidine dihydrochloride (CHX)	µg/mL	5.7 (0.1)	11.1 (2.9)	6.9 (0.1)	4.6 (0.9)
	µM	9.9 (0.2)	19.2 (5.0)	11.9 (0.2)	8.0 (1.6)
		a	a	a	a
Fluconazole (FLC)	µg/mL	3.9 (1.5)	8.1 (1.1)	>1000.0 (0.0)	>1000.0 (0.0)
	µM	12.7 (4.9)	26.4 (3.6)	>3265.1 (0.0)	>3265.1 (0.0)
		b	b	a	a
